# What Eugenics Would Accomplish

**Published:** 1912-09

**Authors:** 


### What Eugenics Would Accomplish.

Eugenics would instill in man higher ideals of living and greater aspirations for doing. It would vastly improve his mental and physical well-being, making him more efficient and his enjoyment of life nearer what was contemplated by the great Architect.

Eugenics would create a new code of ethics, more in conformity to the laws of nature—a religion more responsive to the natural impulses of man and more practical in its betterment of the woeful condition of society. It would create a broader brotherhood of mankind intensely sympathetic to human infirmities and with a quicker conception of what is needed for the good of the whole.

Eugenics would idealize marriage by making it a compact between suitable persons, sexually mated and endowed with a heredity capable of transmitting to their offspring a heritage of physical and mental vigor. It would secure for the marriage state mutual helpfulness and that true satisfaction of human desire that only this union can gratify. It would teach men and women to exercise intelligence in their marital selection, appealing to reason before love blinds the judgment. It would emphasize the responsibility of parenthood, to the end that no union would unthoughtedly bring into the world a child deprived of its inherent rights to a body mentally and physically sound. It would ring the death knell to the old idea that marriages were made in Heaven, to endure forever, regardless of the conditions imposed by nature in the interest of the race. It would clarify the divorce question and purify the morals of society.

Eugenics would, in our generation, greatly lessen the number of the defective, delinquent and dependent class that are now such a burden on society, embarrassing as they do our economical condition, and which have now become a growing menace to our social fabric. It would lessen crime, depravity and poverty; the number of penitentiaries and asylums and, more far-reaching in its results, it would decrease that hereditary strain of nervous instability that is the strongest etiological factor in the morbidity of disease.

Eugenics would clearly show society the deficient points in human progress and the utter impossibility of regenerating the race by our present code of ethics. And more, it would point out the way how these reforms can be brought about by control over the procreative power of man by invoking a simple, safe and scientific surgical procedure.

